# Kidney Function-Dependence of Vitamin K-Status Parameters: Results from the TransplantLines Biobank and Cohort Studies

**DOI:** 10.3390/nu13093069

**Published:** 2021-08-31

**Authors:** Daan Kremer, Dion Groothof, Charlotte A. Keyzer, Coby Eelderink, Tim J. Knobbe, Adrian Post, Marco van Londen, Michele F. Eisenga, TransplantLines Investigators, Leon J. Schurgers, Stefan P. Berger, Martin H. de Borst, Stephan J. L. Bakker

**Affiliations:** 1 Department of Internal Medicine, Division of Nephrology, University of Groningen and University Medical Center Groningen, 9700 RB Groningen, The Netherlands; d.groothof@umcg.nl (D.G.); c.a.keyzer@umcg.nl (C.A.K.); c.eelderink@umcg.nl (C.E.); t.j.knobbe@umcg.nl (T.J.K.); a.post01@umcg.nl (A.P.); m.van.londen@umcg.nl (M.v.L.); m.f.eisenga@umcg.nl (M.F.E.); s.p.berger@umcg.nl (S.P.B.); m.h.de.borst@umcg.nl (M.H.d.B.); s.j.l.bakker@umcg.nl (S.J.L.B.); 2 University Medical Center Groningen Transplant Center, University of Groningen and University Medical Center Groningen, 9700 RB Groningen, The Netherlands; datarequest.transplantlines@umcg.nl; 3 Department of Biochemistry, Cardiovascular Research Institute Maastricht (CARIM), University of Maastricht, 6200 MD Maastricht, The Netherlands; l.schurgers@maastrichtuniversity.nl

**Keywords:** matrix Gla protein, osteocalcin, vitamin K, vascular calcification, kidney transplant, kidney function

## Abstract

High circulating dephosphorylated (dp) uncarboxylated (uc) matrix Gla protein (MGP) and uc osteocalcin (OC) concentrations are regarded as markers of vitamin K-deficiency. However, because MGP and OC are small molecules, they may potentially pass the glomerulus, and their blood concentrations may strongly depend on kidney function. However, many studies with vitamin K-status parameters do not structurally adjust for baseline kidney function, and detailed studies on kidney function-dependence of vitamin K-status markers are lacking. We therefore measured plasma dp-ucMGP using a chemiluminescent assay in 578 kidney transplant recipients (41% females, age 56 ± 13y, 7.5 (3.2 to 13.7)y after transplantation, eGFR 49 ± 17 mL/min/1.73 m^2^) participating in the prospective TransplantLines Cohort Studies. Additionally, dp-carboxylated MGP, ucOC and carboxylated OC were measured using ELISA in plasma of a subgroup of 60 participants. Finally, dp-ucMGP was measured in a separate cohort of 124 kidney transplant recipients before and three months after kidney transplantation. Dp-ucMGP positively correlated with creatinine, cystatin C, and negatively with eGFR (Spearman’s ρ 0.54, 0.60, and −0.54, respectively, *p* < 0.001 for all), and each 10 mL/min/1.73 m^2^ increase in eGFR was associated with a 14.0% lower dp-ucMGP. Additionally, dp-ucMGP strongly declined after kidney transplantation (pretransplantation: 1252 (868 to 1744) pmol/L to posttransplantation: 609 (451 to 914) pmol/L, *p* < 0.001). Proportions of dp-ucMGP over total MGP and ucOC over total OC were not associated with eGFR. This study highlights that dp-ucMGP is strongly associated with kidney function, and that levels strongly decrease after kidney transplantation. We therefore propose adequate adjustment for kidney function, or the use of kidney function-independent parameters such as proportion of uncarboxylated MGP or OC in the assessment of vitamin K-status in clinical practice and research.

## 1. Introduction

Vitamin K and vitamin K-dependent proteins play essential roles in numerous processes in the human body, including roles in hemostasis, bone health, and counteracting vascular calcification [1,2,3,4]. In addition, vitamin K-deficiency is associated with increased cardiovascular risk and mortality [5,6,7]. Consequently, the potential benefits of vitamin K-supplementation are being studied in multiple patient populations [8]. For example, impaired vitamin K-status has been linked to increased vascular calcification, increased cardiovascular risk and mortality in patients with chronic kidney disease, and in kidney transplant recipients [9,10]. However, the majority of stable kidney transplant recipients are reportedly vitamin K-deficient [5]. Consequently, vitamin K has been suggested as a potential modifiable target to alleviate the high cardiovascular risk in this specific population. To identify patients that may benefit from vitamin K-supplementation, and to better understand the roles of vitamin K in health and disease, an adequate assessment of vitamin K-status is essential. Circulating concentrations of vitamin K are infrequently measured in practice, as they mainly reflect recent dietary intake rather than overall vitamin K-status [11]. Therefore, other markers such as dephosphorylated uncarboxylated matrix Gla protein (dp-ucMGP), are often reported as alternative measures of vitamin K-status.

Matrix Gla protein (MGP) is itself an established inhibitor of vascular calcification [1,2]. Its activation requires carboxylation of dp-ucMGP to form dephospho-carboxylated MGP (dp-cMGP), which is then phosphorylated into active mature MGP [12,13]. Because the carboxylation of dp-ucMGP is vitamin K-dependent, high circulating levels of this precursor are considered indicative of poor vitamin K-status [12,13]. It should, however, be realized that MGP is a small molecule (~11 kD) that may pass the glomerulus, and that its blood levels may therefore strongly depend on kidney function [14]. Dp-ucMGP is, in fact, even smaller than cystatin C (~13 kD), which is generally regarded as a marker of kidney function, largely because of this property [15,16]. Another frequently used marker of vitamin K-status is uncarboxylated osteocalcin (ucOC) [17]. This molecule has a molecular weight of less than 6 kD, and may therefore also be susceptible to influences of kidney function [18]. 

Despite the suspected kidney function-dependence of vitamin K-status parameters such as dp-ucMGP, not all studies appropriately adjust for baseline kidney function [19,20]. Unrecognized residual confounding by kidney function may therefore potentially lead to premature or incorrect conclusions on the effects of vitamin K-deficiency [21,22].To the best of our knowledge, detailed studies on the potential influence of kidney function on vitamin K-status parameters are lacking.

Given the significant variation in kidney function, and the high prevalence of vitamin K-insufficiency among kidney transplant recipients [5], this population may serve as an excellent model group to study associations of kidney function with dp-ucMGP, and other suggested markers of vitamin K-status such as ucOC [17]. Therefore, in this study, we aimed to cross-sectionally assess the extent to which dp-ucMGP is associated with kidney function, in a cohort of kidney transplant recipients at least one year after transplantation. In a second cohort of patients undergoing kidney transplant surgery during our study, we prospectively studied changes in plasma dp-ucMGP in patients with available measurements both before and after kidney transplantation, with consequent kidney function improvements after transplantation. Finally, we assessed cross-sectional associations between kidney function and alternative vitamin K-associated markers, including the proportions of uncarboxylated MGP and ucOC over total MGP and OC, respectively, in a subgroup of the first cohort.

## 2. Materials and Methods

### 2.1. Study Population—Cohort 1

Cohort 1 consisted of a total of 578 kidney transplant recipients with available data on circulating dp-ucMGP concentrations. This total cohort consisted of 518 kidney transplant recipients that were enrolled in the ongoing, prospective TransplantLines Biobank and Cohort Study at the time as dp-ucMGP measurements in September 2018 [23,24], and 60 kidney transplant recipients from a previously described study that included patients from 2008 until 2011 [25]. Data on dp-ucMGP were available in both studies, and pooling of both groups ensured a large study population for analyses on dp-ucMGP. Additional data regarding dp-cMGP, ucOC, and cOC were available in the 60 patients enrolled in the second study, which allowed for further exploration of the kidney function-dependence of mentioned alternative vitamin K-status parameters. Both studies included kidney transplant recipients (≥18 years old) with a functional graft, approximately one or longer year after transplantation, who visited the outpatient clinic of the University Medical Center Groningen (UMCG), Groningen, The Netherlands, and had no history of drug or alcohol addiction. All patients provided written informed consent. Both studies were approved by the local institutional review board (UMCG, METc 2008/186, and UMCG, METc 2014/077), and adhere to the WMA Declarations of Helsinki and Istanbul.

### 2.2. Study Population—Cohort 2

The separate, non-overlapping cohort 2 consisted of 124 patients that were also enrolled in the ongoing, prospective TransplantLines Biobank and Cohort Study, but had available data on dp-ucMGP both before and three months after kidney transplantation. These patients were included prior to their transplantation in the University Medical Center Groningen (UMCG), Groningen, The Netherlands, and had an additional study visit three months after transplantation. They had no history of drug or alcohol addiction. A flow diagram visualizing the study population of cohorts 1 and 2 is presented in Figure S1.

### 2.3. Clinical Assessment

The measurements in cohort 1, and the posttransplantation measurements in cohort 2, were performed during a morning visit to the outpatient clinic. The pre-transplantation measurements in cohort 2 were performed immediately prior to the kidney transplantation, at the hospital ward. Blood pressure was measured using a semi-automatic device (Dinamap 1846, Critikon, Tampa, FL, USA) following strict protocols [23]. Diabetes was defined using the American Diabetes Association criteria [26]. Kidney function was assessed by means of creatinine-based eGFR and cystatin C-based eGFR, according to the respective Chronic Kidney Disease Epidemiology Collaboration equations [27,28].

### 2.4. Biochemical Analyses

For cohort 1, and the posttransplantation measurements in cohort 2, blood was drawn after an overnight fasting period of at least 8 hours. For the pretransplantation measurements in cohort 2, blood samples were drawn at the operation theatre, immediately prior to the transplant surgery. For all study subjects, samples were stored at room temperature for a maximum of one hour, centrifuged for ten minutes, and then stored at −80 °C. Plasma dp-ucMGP levels were measured in a single run by the Laboratory of Coagulation Profile (Maastricht, the Netherlands) using the commercially available IVD CE-marked chemiluminescent InaKtif MGP assay on the IDS-iSYS system (IDS, Boldon, UK), which has been described elsewhere [29]. In brief, plasma samples and internal calibrators were incubated using magnetic particles that were coated with murine monoclonal antibodies against dp-MGP, acridinium-labelled murine monoclonal antibodies against ucMGP, and an assay buffer. The magnetic particles were captured using a magnet and washed to remove any unbound analyte. Trigger reagents were added, and the resulting light emitted by the acridinium label was directly proportional to the level of dp-ucMGP in the sample. The assay measuring range was between 300 and 12,000 pmol/L and was linear up to 11,651 pmol/L. The within-run and total variations of this assay were 0.8–6.2% and 3.0–8.2%, respectively. Circulating dp-cMGP concentrations were determined in citrated plasma using a dual-antibody ELISA (VitaK BV, Maastricht, The Netherlands). Circulating ucOC and cOC plasma concentrations were measured using ELISA (Takara Shuzo Co. Ltd., Shiga, Japan). Other routine clinical chemistry assays were performed using automated and validated routine laboratory methods (Roche Diagnostics, Basel, Switzerland).

### 2.5. Statistical Analyses

Baseline data are presented separately for cohort 1 and cohort 2, and shown as mean ± standard deviation for normally distributed data, as median (interquartile range) for non-normally distributed data, and as number (percentage) for nominal data. The associations of vitamin K-antagonist use and eGFR with plasma dp-ucMGP concentration was assessed using linear regression analyses, where dp-ucMGP was log_2_ transformed to fulfill the assumptions of linear regression with regard to homoscedasticity and distribution of the residuals. Change in dp-ucMGP depending on vitamin K-antagonist use and eGFR increase are given as percentages. Associations of creatinine-based eGFR with dp-ucMGP were assessed in cohort 1 using linear regression analyses, and were visualized using scatter plots. The linear regression coefficient is given as standardized beta (St. β), which indicates the number of standard deviations in dp-ucMGP changes per standard deviation increase in eGFR. The change in dp-ucMGP after kidney transplantation (with consequent improvements in kidney function) was visualized using boxplots of dp-ucMGP before and three months after kidney transplantation, where significance of differences between dp-ucMGP before and after transplantation was assessed using the Wilcoxon signed rank test, and equality of variances between the groups was assessed using Levene’s test. In sensitivity analyses, these analyses were repeated after exclusion of patients with vitamin K-antagonist use either before or after transplantation.

In the subgroup of 60 kidney transplant recipients with available data on dp-ucMGP, dp-cMGP, ucOC, cOC, and proportions of uncarboxylated MGP and OC, the correlations of these vitamin K-status-associated parameters with kidney function-associated parameters were visualized using a correlation heat map, where Spearman’s rank correlation coefficient was multiplied by 100 to improve readability (e.g., a Spearman’s ρ of 0.27 was presented as 27). The Bonferroni method was used to adjust for multiple testing, and statistically insignificant correlations were blanked. The linear associations of eGFR with the previously mentioned vitamin K-status-associated parameters were visualized using scatter plots, with unadjusted R^2^ presented in the figure to estimate the proportions of variation in dp-ucMGP, dp-cMGP, ucOC, cOC, and proportions of uncarboxylated MGP and OC over total MGP and OC, respectively, that may be explained by kidney function. In additional multivariable linear regression analyses, the associations of vitamin K-antagonist use and eGFR with plasma dp-ucMGP and ucOC concentrations and with proportion of ucMGP and ucOC over total MGP and OC, respectively, were assessed using linear regression analyses, where variables were log_2_ transformed if necessary to fulfill the assumptions of linear regression with regard to homoscedasticity and distribution of the residuals.

All data were analyzed using R version 3.5.1 (Vienna, Austria). For all analyses, a two-sided *p*-value < 0.05 was considered statistically significant, unless mentioned otherwise.

## 3. Results

### 3.1. Baseline Characteristics

Cohort 1 consisted of a total of 578 kidney transplant recipients (237 females, 41%). Mean age was 56 ± 13 years, and patients were at median 7.5 (3.2 to 13.7) years after transplantation. Mean eGFR was 49 ± 17 mL/min/1.73 m^2^, and 14% of patients used vitamin K-antagonists. Median circulating dp-ucMGP concentrations were 671 (495 to 1076) pmol/L, with median concentrations of 1842 (1352 to 2644) pmol/L among vitamin K-antagonists users, and 606 (478 to 838) pmol/L among non-users, respectively. In a subgroup of 60 kidney transplant recipients of this cohort [25], we also measured circulating concentrations of ucOC and carboxylated forms of MGP and OC, which allowed for calculation of the proportions of dp-ucMGP and ucOC over total circulating concentrations, which were 21 (16 to 25)% and 15 (9 to 31)%, respectively. 

The non-overlapping prospective cohort 2 consisted of a total of 124 kidney transplant recipients (45 females, 36%) with measurements both prior to and three months after transplantation. Prior to transplantation, the included patients had poor kidney function, which improved after transplantation to an eGFR of 49 ± 14 mL/min/1.73 m^2^ at three months after transplantation. In this group, 12 patients (10%) used vitamin K-antagonists prior to transplantation, although this use was temporarily discontinued three days prior to the transplantation in nine of these patients, in accordance with the local guidelines for antithrombotic agents prior to surgery. In total, 14% of patients used vitamin K-antagonists three months after transplantation. More extensive characteristics of both cohorts are presented in Table 1.

### 3.2. Association of dp-ucMGP with Kidney Function in Cohort 1

In cohort 1, linear regression analyses confirmed that the use of vitamin K-antagonists was indeed associated with higher plasma dp-ucMGP concentrations, as presented in Table 2. Addition of eGFR as an independent variable significantly improved the linear regression model (R^2^ increase from 0.348 to 0.531, *p* < 0.001). These analyses showed that, independent of vitamin K-antagonist use, each 10 mL/min/1.73 m^2^ increase in eGFR was associated with a 14.0% decrease in dp-ucMGP.

**Figure 1:**
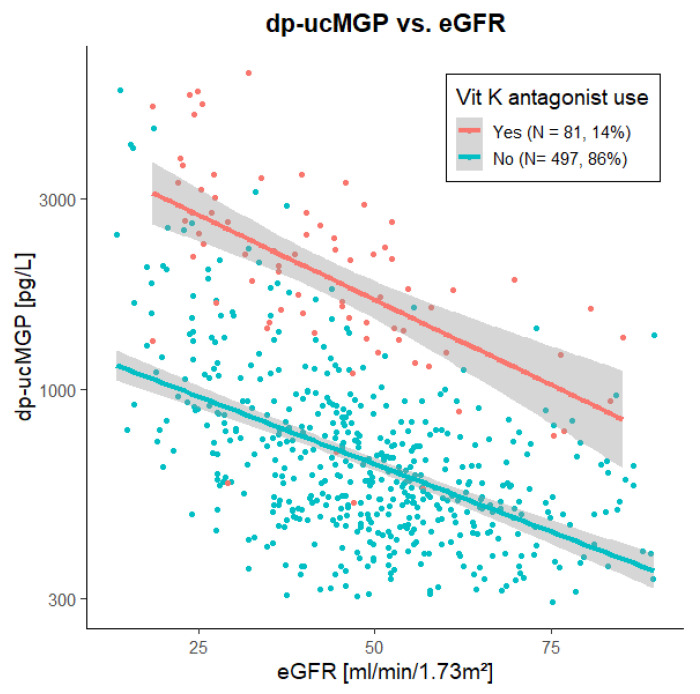
Scatter plot and linear association of dp-ucMGP with eGFR in 578 kidney transplant recipients with and without vitamin K-antagonist use. Dp-ucMGP was strongly associated with eGFR in both vitamin K-antagonist users (St. β −0.584, *p* < 0.001) and non-users (St. β −0.518, *p* < 0.001). Abbreviations: dp-ucMGP, dephosphorylated matrix Gla protein; eGFR, creatinine-based estimated glomerular filtration rate; Vit K, vitamin K.

The strong association of eGFR with circulating dp-ucMGP in both vitamin K-antagonist users and non-users (vitamin K-antagonist users: St. β −0.584, *p* < 0.001; non-users: St. β −0.518, *p* < 0.001) is visualized in Figure 1.

### 3.3. Changes in of dp-ucMGP after Kidney Transplantation in Cohort 2

**Figure 2:**
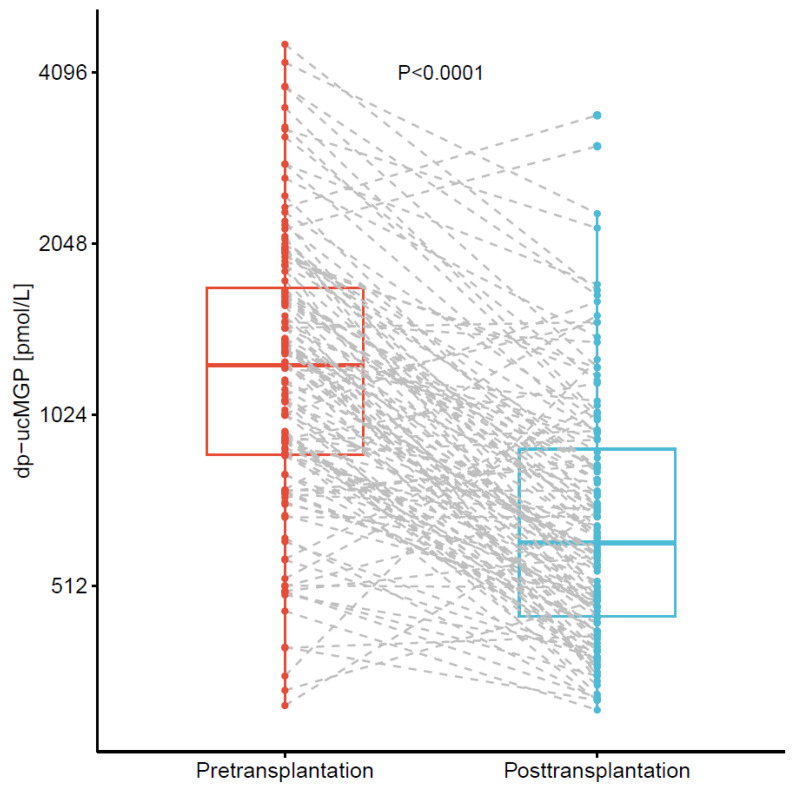
Plasma dp-ucMGP before and three months after kidney transplantation in 124 kidney transplant recipients from cohort 2. Dp-ucMGP plasma concentrations were strongly decreased at three months after transplantation, compared to immediately prior to transplantation (median decrease 50% (29% to 63%)). Abbreviations: dp-ucMGP, dephosphorylated uncarboxylated matrix Gla protein.

Dp-ucMGP plasma concentrations decreased drastically by median 50% (29% to 63%) after kidney transplantation, as presented in Figure 2 (median pretransplantation: 1252 (868 to 1744) pmol/L to median posttransplantation: 609 (451 to 914) pmol/L, *p* < 0.001). In sensitivity analyses, this decrease was of similar magnitude (median 52% (32% to 63%)) after exclusion of 18 patients (15%) with vitamin K-antagonist use either before or three months after transplantation (median pretransplantation: 1172 (790 to 1633) pmol/L to median posttransplantation: 555 (426 to 747) pmol/L, *p* < 0.001). 

In total, only six patients (5%) showed increases in dp-ucMGP plasma concentrations of at least 500 pmol/L after transplantation. Four of these patients used vitamin K-antagonists at three months after transplantation, the use of which had been temporarily discontinued three days before transplantation because of the planned surgery. 

### 3.4. Correlations and Associations of dp-ucMGP, dp-cMGP, ucOC, and cOC in Cohort 1

**Figure 3:**
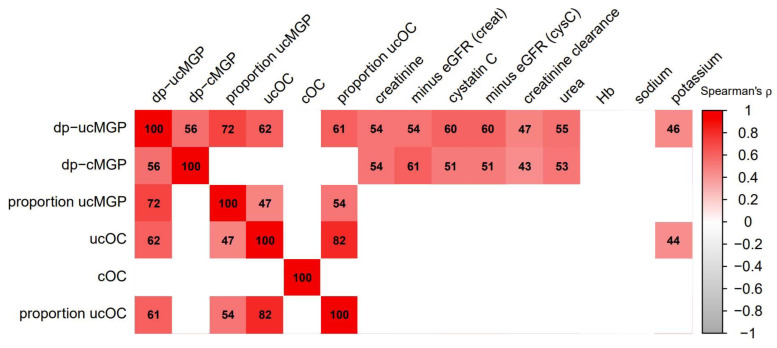
Correlation heat map of vitamin K-associated and kidney function-associated parameters, in a subgroup of 60 kidney transplant recipients from cohort 1. Numbers indicate Spearman’s rank-based coefficients multiplied by 100. *p*-values were multiplied by 90 to adjust for multiple testing (following the Bonferroni method), and the tiles of insignificant correlations are blanked. Abbreviations: dp, dephosphorylated; eGFR, estimated glomerular filtration rate; MGP, matrix Gla protein; OC, osteocalcin; (u)c, (un)carboxylated.

As presented in Figure 3, both dp-ucMGP and dp-cMGP strongly correlated with parameters of kidney function, including creatinine, cystatin C, and GFR estimates (Spearman’s ρ > 0.50 for all) in the subgroup of patients with available data on additional vitamin K-associated parameters from cohort 1. There were no significant correlations of proportion of uncarboxylated MGP with any of the mentioned kidney function parameters. Neither ucOC nor cOC were significantly correlated with kidney function, and neither was the proportion of uncarboxylated OC.

**Figure 4:**
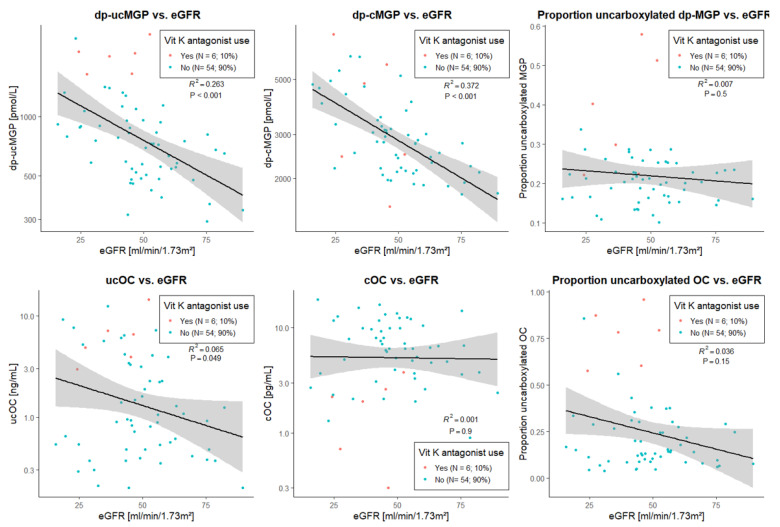
Visual presentation of the univariable linear association of uncarboxylated, carboxylated and proportion uncarboxylated matrix Gla protein and osteocalcin with eGFR, in a subgroup of 60 kidney transplant recipients from cohort 1. In brief, both dp-ucMGP and dp-cMGP were strongly negatively associated with eGFR, whereas the negative association of ucOC with eGFR was less pronounced. No statistically significant associations of cOC, proportion of uncarboxylated dp-MGP and proportion of uncarboxylated OC were found with eGFR. Abbreviations: dp-ucMGP, dephosphorylated uncarboxylated matrix Gla protein; dp-cMGP, dephosphorylated carboxylated matrix Gla protein; eGFR, creatinine-based estimated glomerular filtration rate; ucOC, uncarboxylated osteocalcin; cOC, carboxylated osteocalcin.

The linear associations of eGFR with the mentioned vitamin K-associated parameters are visualized in Figure 4, where linear regression analyses also indicate significant associations of eGFR with dp-ucMGP and dp-cMGP (St. β~ −0.5 for both, *p* < 0.001), but a much weaker association with the proportion of uncarboxylated MGP (St. β −0.256, *p* = 0.049), and no significant associations with the osteocalcin parameters (*p* > 0.1 for all). These associations remained materially unchanged regardless of adjustments for age, sex, and use of vitamin K-antagonists (for dp-ucMGP: St. β −0.414, *p* < 0.001; for dp-cMGP: St. β −0.595, *p* < 0.001).

Linear regression analyses also confirmed that, also in the subgroup of 60 patients from cohort 1, eGFR was significantly associated with dp-ucMGP independent of vitamin K-antagonist use (Table S1). Similarly, there appeared to be a trend towards lower plasma ucOC with higher eGFR, but this association did not reach statistical significance (estimated change in ucOC per 10 mL/min/1.73 m^2^ increase in eGFR: −12.3%; *p* = 0.132, Table S2). In contrast, eGFR was not associated with the proportion of dp-ucMGP over total MGP (*p* = 0.924, Table S3), and the proportion of ucOC over total OC (*p* = 0.761, Table S4). These analyses also confirmed that use of vitamin K-antagonists was associated with strongly increased dp-ucMGP, ucOC, proportion of uncarboxylated MGP over total MGP, and proportion of uncarboxylated OC over total OC.

## 4. Discussion

In this study, we showed that circulating dp-ucMGP concentration is strongly associated with kidney function. This finding was supported by the evident decrease in dp-ucMGP shortly after kidney transplantation, compared to prior to the transplantation. These findings call for prudence in assessment of vitamin K-status using only dp-ucMGP, without taking into account kidney function. In contrast, we found no associations of kidney function with alternative markers of vitamin K-status, such as proportion of uncarboxylated MGP and proportion of uncarboxylated OC, suggesting that these measures may have potential as kidney function-independent measures of vitamin K-status.

Vitamin K plays key roles in numerous physiological processes in the human body. Its roles in hemostasis and bone health are well established, and its roles in counteracting vascular calcification are increasingly recognized [1,2,3,4]. Vitamin K-deficiency is strongly associated with increased cardiovascular risk and mortality, and is common in patients with kidney disease [5,6,7,30,31]. Consequently, the effects of vitamin K supplementation have been extensively studied in multiple patient populations [8]. Adequate assessment of vitamin K-status is necessary to identify patients that may benefit from vitamin K supplementation, and to assess the role of vitamin K in health and disease in clinical research. Vitamin K blood concentrations are infrequently measured in clinical practice, because circulating concentrations mainly reflect recent dietary intake, rather than overall vitamin K-status [11]. Other markers such as dp-ucMGP are therefore frequently reported as an alternative to assess vitamin K-status, for example in kidney transplant recipients, patients with chronic kidney disease, and COVID-19 patients [5,19,20]. Our analyses confirmed that the use of vitamin K-antagonists is associated with strongly increased dp-ucMGP concentrations, which is reassuring with regard to the validity of the used assay. However, our study also shows that kidney function is strongly associated with dp-ucMGP concentrations, with an estimated 14.0% decrease in dp-ucMGP for each 10 mL/min/1.73 m^2^ increase in creatinine-based eGFR. A previous study has shown a kidney function-independent kidney fractional extraction of ~12.8% of circulating MGP [32]. This seemingly contradicts our finding of a strong association of eGFR with both concentrations of dp-ucMGP and dp-cMGP. However, unfortunately, the previous study on kidney fractional extraction of circulating MGP showed no associations or correlations of eGFR or other clinically used kidney function parameters with circulating MGP concentrations, with the exception of kidney fractional extraction of MGP. 

The strong association of kidney function with dp-ucMGP may be explained by the small molecular size of dp-ucMGP, which consists of only 84 amino acids with a molecular weight of approximately 11 kDa [14], which is smaller than, e.g., cystatin C [15,16]. Like cystatin C, it is therefore plausible that a large proportion of circulating dp-ucMGP is filtered by the glomerulus, and that its concentrations do not only reflect vitamin K-status, but also are strongly dependent on kidney function. This notion is supported by the strong decrease in dp-ucMGP observed in our study shortly after kidney transplantation. A small proportion of the patients in our study (six patients, 5% of total population) showed evidently elevated dp-ucMGP concentrations after transplantation. In four of these six patients, the use of vitamin K-antagonists in these patients had been temporarily discontinued three days prior to transplantation because of the planned surgery; the re-initiation of vitamin K-antagonist in these patients after surgery explains the increased dp-ucMGP after transplantation despite improvements in kidney function in these patients. 

Associations of dp-ucMGP with kidney function and incident chronic kidney disease have been previously addressed [19,33,34,35]. In these studies, kidney function declines were generally regarded as a result of vitamin K-deficiency, as deduced from high circulating dp-ucMGP concentrations. However, our study underlines that decreased kidney function may largely be a cause of increased circulating dp-ucMGP concentrations, rather than the result of vitamin K-deficiency. Indeed, a reported association between dp-ucMGP and incident chronic kidney disease was recently shown to be driven by the baseline effects of kidney function in a different population-based cohort [19,21], and our results support speculation that other prospective associations of dp-ucMGP with outcome may also be the result of impaired kidney function, rather than vitamin K-deficiency. 

An alternative marker of vitamin K-deficiency is ucOC. This protein is secreted by osteoblasts and undergoes post-translational vitamin K-dependent carboxylation processes similar to MGP [6,18]. However, osteocalcin also is a small protein [18], and the association with kidney function also reached statistical significance in linear regression analyses. Our results show that ucOC was much less strongly associated with glomerular filtration rate compared to dp-ucMGP. This may seem surprising, given the molecular weight of ucOC, which is smaller than that of dp-ucMGP. However, the negatively charged protein surface of ucOC may explain this finding, because negatively charged molecules are less able to cross the negatively charged glomerular membrane [36]. Although the association of kidney function with ucOC was weaker compared to dp-ucMGP, the association may still be important in larger studies. Therefore, there remains a need for alternative kidney function-independent parameters of vitamin K-status. We propose that these may be found in proportions of uncarboxylated MGP or OC, which were not associated with kidney function in our study. The use of these kidney function-independent parameters to assess vitamin K-status should be replicated in larger cross-sectional and prospective studies. 

With this study, we underline that generally accepted markers of vitamin K-deficiency, may be strongly affected by kidney function. Hence, some associations of dp-ucMGP with increased risk of detrimental outcome may be confounded by kidney function [19,20,21,22], which had not been accounted for. We can by no means disprove that vitamin K-deficiency may be detrimental to patient outcome, and can also not disprove that vitamin K may have renoprotective effects. We do, however, want to raise awareness among clinicians to adequately take kidney function into account when assessing vitamin K-status, and urge researchers to consistently adjust for baseline kidney function when reporting associations of vitamin K-status parameters with clinical outcome, or to use alternative parameters, such as proportion of uncarboxylated osteocalcin, to assess vitamin K-status. 

The strengths of this study are the large cohort of kidney transplant recipients, with large kidney function variability, rendering it a suitable population for studying associations of kidney function with vitamin K-status parameters. Future studies are necessary to confirm that these strong associations of kidney function with vitamin K-status parameters are also present in other patient populations, such as patients with chronic kidney disease. An additional strength of this study is the unique availability of serial measurements of dp-ucMGP both before and shortly after transplantation, which highlights, for the first time, that circulating dp-ucMGP concentrations decrease drastically after within-patient kidney function improvements. Finally, the additional measurements in dp-cMGP, ucOC, and cOC in a subgroup of included patients provided further insight into the associations of kidney function with these parameters, and highlight the potential of proportions of uncarboxylated MGP and osteocalcin as kidney-function independent markers of vitamin K-status. It must be noted, however, that data on these additional vitamin K-status parameters were available only in a subgroup of 60 kidney transplant recipients. Future studies may therefore replicate these findings in different cohorts. The finding that vitamin K-antagonist use was indeed associated with strongly increased dp-ucMGP and ucOC concentrations is reassuring with regard to consistency and validity of the used assays. An important limitation to the current study is that, due to its observational design, no conclusions regarding causality can be drawn from these results. In addition, we were unable to assess the associations of dietary patterns with dp-ucMGP prior to and after transplantation, although changes in diet after transplantation may account for some part of the decrease in dp-ucMGP in cohort 2. Future studies may assess the value of alternative markers of vitamin K-status, including growth arrest specific protein 6, Gla-rich protein, and PIVKA-II [6,37]. Such studies may also prospectively assess and compare the mentioned molecules as markers of cardiovascular risk, after adjustment for kidney function.

In conclusion, this study highlights that dp-ucMGP is strongly associated with kidney function, and that levels strongly decrease shortly after kidney transplantation. We therefore propose adequate adjustment for kidney function, or the use of kidney function-independent parameters, such as proportions of uncarboxylated matrix Gla protein or osteocalcin, in assessment of vitamin K-status, especially in patients with impaired kidney function.

## Figures and Tables

**Table 1:** Population characteristics at baseline for both cohorts.

	Cohort 1 *N* = 578	Cohort 2 *N* = 124 with Serial Measurements	
Time of Blood Sampling	7.5 (3.2 to 13.7) Years after Transplantation	At Transplantation	~3 Months after Transplantation
dp-ucMGP, pmol/L	671 (495 to 1076)	1252 (868 to 1744)	609 (451 to 914)
Clinical characteristics			
Female sex, *n* (%)	237 (41)	45 (36)	-
Age, years	56 (13)	53 (14)	-
Height, cm	173 (10)	175 (10)	-
Weight, kg	82 (16)	81 (15)	82 (15)
Systolic blood pressure, mmHg	135 (17)	140 (19)	133 (14)
Diabetes, *n* (%)	146 (25)	17 (14)	32 (26)
Laboratory measurements			
Hemoglobin, mmol/L	8.5 (1.1)	7.5 (1.0)	7.9 (1.1)
Sodium, mmol/L	140.1 (2.7)	139.0 (2.5)	140.0 (2.4)
Potassium, mmol/L	4.0 (0.4)	4.6 (0.7)	4.0 (0.4)
Creatinine, µmol/L	129 (108 to 163)	595 (441 to 776)	130 (111 to 161)
eGFR, mL/min/1.73 m^2^	49 (17)	-	49 (14)
Urea, mmol/L	8.8 (6.7 to 12.0)	22.3 (16.8 to 28.3)	8.4 (6.4 to 10.4)
HbA1c, mmol/mol	39 (36 to 45)	36 (34 to 40)	40 (36 to 46)
Medication use			
Vitamin K-antagonists, *n* (%)	81 (14)	12 (10) *Discontinued in 9 (7) prior to planned surgery*	17 (14)
Prednisolone, *n* (%)	559 (97)	24 (19)	123 (99)
Calcineurin inhibitor, *n* (%)	414 (72)	-	123 (99)
Proliferation inhibitor, *n* (%)	476 (82)	-	117 (94)
mTOR inhibitor, *n* (%)	20 (3)	-	4 (3)
Other vit. K-associated parameters	Subgroup (*N* = 60)		
dp-cMGP, pmol/L	2787 (2169 to 3688)		
Proportion uncarboxylated MGP, %	21 (16 to 25)		
ucOC, pmol/L	1.07 (0.49 to 3.95)		
cOC, pmol/L	6.2 (3.2 to 9.9)		
Proportion uncarboxylated OC, %	15 (9 to 31)		

Normally distributed data are presented as mean ± standard deviation, skewed data as median (interquartile range), and categorical data as number (valid percentage). Diabetes was defined according to the American Diabetes Association criteria. Abbreviations: dp, dephosphorylated; eGFR, estimated glomerular filtration rate as calculated using the creatinine-based CKD-EPI formula; HbA1c, hemoglobin A1c; MGP, matrix Gla protein; OC, osteocalcin; (u)c, (un)carboxylated.

**Table 2:** Linear regression analyses with log_2_ plasma dp-ucMGP concentration as dependent variable in 578 kidney transplant recipients from cohort 1.

	Variable	Change in dp-ucMGP	T-Value	*p*-Value	Model R^2^
Model 1	Vitamin K-antagonist use, yes vs. no	+181.3%	-	<0.001	0.348
Model 2	eGFR, per 10 mL/min/1.73 m^2^ increase	−15.4%	-	<0.001	0.224
Model 3	Vitamin K-antagonist use, yes vs. no	+165.1%	19.4	<0.001	0.531
	eGFR, per 10 mL/min/1.73 m^2^ increase	−14.0%	−15.0	<0.001	

Change in dp-ucMGP indicates the percentage of change in dp-ucMGP for vitamin K-antagonist use, and eGFR (per 10 mL/min/1.73 m^2^ increase), where + indicates increasing and—indicate decreasing dp-ucMGP. T-values indicate the size of the difference relative to the variation in the data, thus allowing for comparison of the strengths of the associations of vitamin K-antagonist use and eGFR in model 3. Addition of eGFR significantly improved the model (*p* < 0.001). Abbreviations: eGFR: creatinine-based estimated glomerular filtration rate as calculated using the CKD-EPI formula.

## Data Availability

Data is available upon reasonable request to the corresponding author.

## Supplementary Materials

The following are available online at https://www.mdpi.com/article/10.3390/nu13093069/s1, Figure S1: Diagram visualizing the flow of participants through the study. Tables S1 to S4: Linear regression analyses with plasma dp-ucMGP (S1), plasma ucOC (S2), proportion dp-ucMGP over total MGP (S3), and proportion ucOC over total OC (S4) in the subgroup of 60 patients with data on dp-cMGP, ucOC and cOC from cohort 1.
