# Supplementary material for: Kidney Function-Dependence of Vitamin K-Status Parameters: Results from the TransplantLines Biobank and Cohort Studies
Source: Nutrients. 2021 Aug 31;13(9):3069. doi: 10.3390/nu13093069 (PMC8467091; doi:10.3390/nu13093069)
Supplement: Supplementary file 1 [file nutrients-13-03069-s001.zip › nutrients-1348911-SI.pdf]

nutrients

MDPI

Supplementary

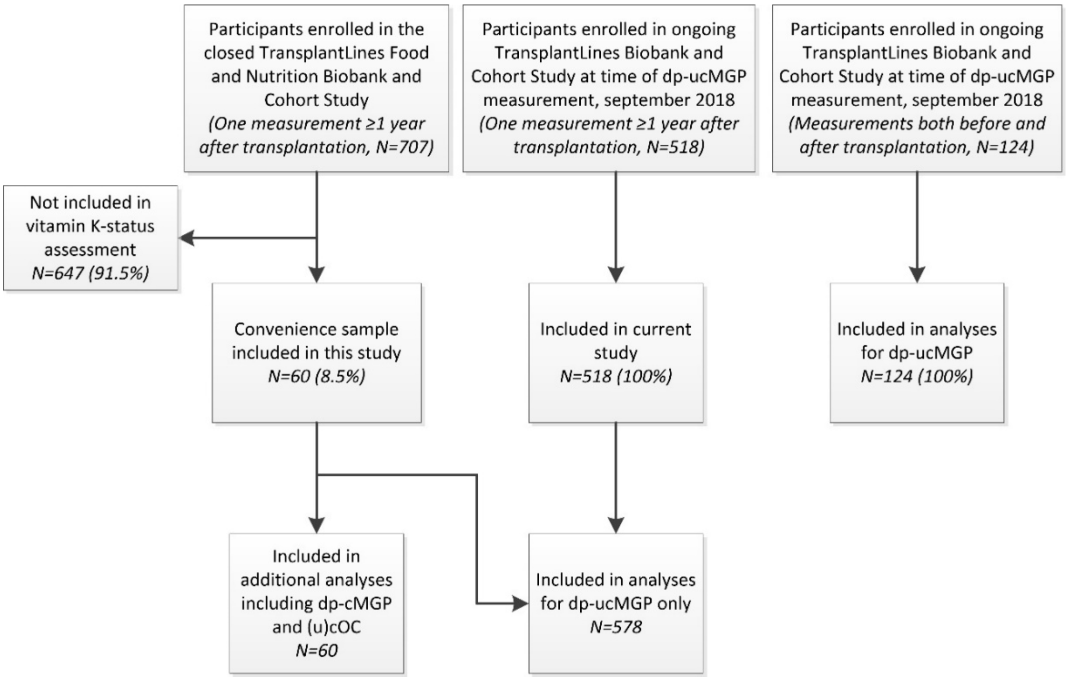

Figure S1. Diagram visualizing the flow of participants through the study.

Table S1. Linear regression analyses with plasma dp-ucMGP as the dependent variable in a subgroup of 60 kidney transplant recipients from cohort 1.

	Variable	Change in dp-ucMGP	T-value	P-value	Model R^2
**Model 1**	Vitamin K-antagonist use, yes vs. no	+ 185.4%	-	<0.001	0.372
**Model 2**	eGFR, per 10 ml/min/1.73m^2 increase	- 15.0%	-	<0.001	0.251
**Model 3**	Vitamin K-antagonist use, yes vs. no	+ 149.7%	5.80	<0.001	0.537
	eGFR, per 10 ml/min/1.73m^2 increase	- 12.3%	-4.50	<0.001	

Change in dp-ucMGP indicates the percentage of change in dp-ucMGP for vitamin K-antagonist use, and eGFR (per 10 mL/min/1.73m^2 increase), where + indicates increasing and – indicate decreasing dp-ucMGP. T-values indicate the size of the difference relative to the variation in the data, thus allowing for comparison of the strengths of the associations of vitamin K-antagonist use and eGFR in model 3. Abbreviations: eGFR: creatinine-based estimated glomerular filtration rate as calculated using the CKD-EPI formula.

Table S2. Linear regression analyses with plasma ucOC as the dependent variable in a subgroup of 60 kidney transplant recipients from cohort 1.

	Variable	Change in ucOC	T-value	P-value	Model R^2
**Model 1**	Vitamin K-antagonist use, yes vs. no	+ 404.4%	-	<0.001	0.175
**Model 2**	eGFR, per 10 ml/min/1.73m^2 increase	- 16.7%	-	0.049	0.065
**Model 3**	Vitamin K-antagonist use, yes vs. no	+ 341.5%	3.20	0.002	0.208
	eGFR, per 10 ml/min/1.73m^2 increase	- 12.3%	-1.53	0.132	

Change in dp-ucMGP indicates the percentage of change in dp-ucMGP for vitamin K-antagonist use, and eGFR (per 10 mL/min/1.73m² increase), where + indicates increasing and – indicate decreasing dp-ucMGP. T-values indicate the size of the difference relative to the variation in the data, thus allowing for comparison of the strengths of the associations of vitamin K-antagonist use and eGFR in model 3. Abbreviations: eGFR: creatinine-based estimated glomerular filtration rate as calculated using the CKD-EPI formula.

Table S3. Linear regression analyses with proportion of dp-ucMGP over total MGP as the dependent variable in a subgroup of 60 kidney transplant recipients from cohort 1.

	Variable	Change in proportion uncarboxylated MGP	T-value	P-value	Model R^2
**Model 1**	Vitamin K-antagonist use, yes vs. no	+ 77.5%	-	<0.001	0.267
**Model 2**	eGFR, per 10 ml/min/1.73m² increase	- 0.0%	-	0.512	0.007
**Model 3**	Vitamin K-antagonist use, yes vs. no	+ 77.9%	4.49	<0.001	0.267
	eGFR, per 10 ml/min/1.73m² increase	- 0.0%	0.10	0.924	

Change in dp-ucMGP indicates the percentage of change in dp-ucMGP for vitamin K-antagonist use, and eGFR (per 10 mL/min/1.73m² increase), where + indicates increasing and – indicate decreasing dp-ucMGP. T-values indicate the size of the difference relative to the variation in the data, thus allowing for comparison of the strengths of the associations of vitamin K-antagonist use and eGFR in model 2. Abbreviations: eGFR: creatinine-based estimated glomerular filtration rate as calculated using the CKD-EPI formula.

Table S4. Linear regression analyses with proportion ucOC over total OC as the dependent variable in a subgroup of 60 kidney transplant recipients from cohort 1.

	Variable	Change in proportion uncarboxylated OC	T-value	P-value	Model R^2
**Model 1**	Vitamin K-antagonist use, yes vs. no	+ 396.8%	-	<0.001	0.341
**Model 2**	eGFR, per 10 ml/min/1.73m² increase	- 9.2%	-	0.146	0.036
**Model 3**	Vitamin K-antagonist use, yes vs. no	+ 376.1%	5.22	<0.001	0.348
	eGFR, per 10 ml/min/1.73m² increase	- 4.1%	-0.76	0.761	

Change in dp-ucMGP indicates the percentage of change in dp-ucMGP for vitamin K-antagonist use, and eGFR (per 10 mL/min/1.73m² increase), where + indicates increasing and – indicate decreasing dp-ucMGP. T-values indicate the size of the difference relative to the variation in the data, thus allowing for comparison of the strengths of the associations of vitamin K-antagonist use and eGFR in model 3. Abbreviations: eGFR: creatinine-based estimated glomerular filtration rate as calculated using the CKD-EPI formula.
